# Reduced fetal growth velocities and the association with neonatal outcomes in appropriate-for-gestational-age neonates: a retrospective cohort study

**DOI:** 10.1186/s12884-018-2167-5

**Published:** 2019-01-15

**Authors:** M. L. E. Hendrix, S. M. J. van Kuijk, A. W. D. Gavilanes, D. Kramer, M. E. A. Spaanderman, S. Al Nasiry

**Affiliations:** 1 Department of Obstetrics & Gynaecology, GROW School of Oncology and Developmental Biology, Maastricht University Medical Centre (MUMC), PO Box 5800, 6202 AZ Maastricht, The Netherlands; 2 Department of Clinical Epidemiology and Medical Technology Assessment (KEMTA), Maastricht, University Medical Centre (MUMC), Maastricht, The Netherlands; 3 Department of Paediatrics, Maastricht University Medical Centre (MUMC), Maastricht, The Netherlands; 4 Department of Translational Neuroscience, School for Mental Health and Neuroscience (MHeNS), Maastricht University, Maastricht, The Netherlands; 5 Institute of Biomedicine, Facultad de Ciencias Médicas, Universidad Católica de Santiago de Guayaquil, Guayaquil, Ecuador

**Keywords:** Fetal growth restriction, Abdominal circumference velocity, Neonatal outcome, Ultrasound, Appropriate-for-gestational-age neonates

## Abstract

**Background:**

Fetal growth restriction is, despite advances in neonatal care and uptake of antenatal ultrasound scanning, still a major cause of perinatal morbidity. Neonates with birth weight > 10th percentile are assumed to be appropriate-for-gestational-age (AGA), although many are at increased risk of perinatal morbidity, because of undetected mild restriction of growth potential. We hypothesized that within AGA neonates, reduced fetal growth velocities are associated with adverse neonatal outcome.

**Methods:**

A retrospective cohort study of singleton pregnancies, in the Maastricht University Medical Centre (MUMC) between 2010 and 2016. Women had two fetal biometry scans (18–22 weeks and 30–34 weeks of gestational age) and delivered a newborn with a birth weight between the 10th–80th percentile.

Differences in growth velocities of the abdominal circumference (AC), biparietal diameter (BPD), head circumference (HC) and femur length (FL) were compared between the suboptimal AGA (sAGA) (birth weight centiles 10–50) and optimal AGA (oAGA) (birth weight centiles 50–80) group. We assessed the association between velocities and neonatal outcomes.

**Results:**

We included 934 singleton pregnancies. In the suboptimal AGA group, fetal growth velocities were lower (in mm/week): AC 10.72 ± 1.00 vs 11.23 ± 1.00 (*p* < .001), HC 10.50 ± 0.80 vs 10.68 ± 0.77 (*p* = 0.001), BPD 3.01 ± 0.28 vs 3.08 ± 0.27 (*p* < .0001) and FL 2.47 ± 0.21 vs 2.50 ± 0.22 (*p* = 0.014), compared to the optimal AGA group. Neonates with an adverse neonatal outcome had significantly lower growth velocities (in mm/week) of: AC 10.57 vs 10.94 (*p* = 0.034), HC 10.28 vs 10.59 (*p* = 0.003) and BPD 2.97 vs 3.04 (*p* = 0.043) compared to those with normal outcome. An inverse association was observed between the AC velocity and a composite adverse neonatal outcome (OR) = 0.667 (95%CI 0.507–0.879, *p* = 0.004), and between the AC velocity and neonates with NICU stay (OR) = 0.733 (95%CI 0.570–0.942, *p* = 0.015). Neonates with a birthweight lower than expected (based on the abdominal circumference at 20 weeks) had significantly more composite adverse neonatal outcomes 8.5% vs 5.0% (*p* = 0.047), NICU stays 9.6% vs 3.8% (*p* < .0001) and hospital stays 44.4% vs 35.6% (*p* = 0.006).

**Conclusions:**

Appropriate-for-gestational-age neonates are a heterogeneous group with some showing suboptimal fetal growth. Abnormal fetal growth velocities, especially abdominal circumference velocity, are associated with adverse neonatal outcome and can potentially improve the detection of mild growth restriction when used in multivariate models.

## Key message

There is a subgroup within appropriate-for-gestational age neonates, who are at increased risk of perinatal morbidity. The abdominal circumference velocity could be a detector for this group with mild growth restriction.

## Background

Fetal growth restriction (FGR) is one of the most frequently encountered problems in modern obstetrics, with a major impact on perinatal mortality and morbidity [1]. FGR is a controversial and complex entity, due to its multifactorial aetiology and the unclear link between its pathophysiology and its current definitions.

Literature has often interchangeably used the terms ‘intrauterine growth retardation’, ‘intrauterine growth restriction’ (both given the acronym IUGR) and fetal growth restriction (FGR), to indicate abnormally small fetus suspected antenatally, based on ultrasound parameters of fetal growth, most commonly expected fetal weight (EFW) and abdominal circumference (AC) [2]. We prefer to use the moderner term FGR being more specific, i.e. pertaining to the real problem, the fetus, and not to other intrauterine tissues, and additionally, not having the negative connotation of the word “retardation”. Traditionally, EFW and AC are plotted against population curves, on which there is no consensus and are the subject of ongoing debate, and IUGR is most commonly defined as EFW of AC values below the 3rd, 5th or 10th centile [3]. Small for gestational age (SGA) is the term used postnatally to describe a newborn with abnormally low birth weight for a specific gestational period, using variable cut-off points in literature: 3rd, 5th or 10th centile. The term SGA has also been inappropriately used antenatelly to define fetus with mild to moderate “smallness” (EFW or AC between 3rd-10th centile.

These definitions are based on cut off points and statistical assumptions derived from epidemiological data, rather than being derived from physiopathological mechanisms. It is conceivable that the mechanism restricting fetal growth can have variable severity, leading in mild cases to a decrease in growth velocity but not enough for birth weight to drop below the 10th centile. Hence, these neonates with restricted growth potential are falsely labelled as appropriate-for-gestational age (AGA) according to many commonly used population charts.

Unlike severe early FGR, mild and late onset FGR in the AGA group is difficult to diagnose and more elusive to currently used predictive tests [4, 5]. Fetuses with the lowest birth weight centiles have a significantly higher risk of perinatal mortality compared to the 50-90th centile [6]. This largely undetected type of growth restriction is responsible for a significant percentage of stillbirths and neonates with adverse neonatal outcome [7, 8]. Routine third trimester biometry scans could improve the detection of FGR in the AGA group [9]. However, such scans take a ‘snapshot‘ approach to fetal growth and fail to take into account biological variation influenced by the genetic background and also the concept of suboptimal fetal growth being a dynamic process. A better approach is to consider the difference in a growth parameter between two antenatal time points, corrected for the genetically determinded growth potential of an individial fetus, tentatively expressed as ‘growth velocity’ [10]. This individualised growth potential can describe a type of growth restriction characterized by a drop in growth potential without necessarily being overtly small [11]. We hypothesized that within the AGA group, reduced fetal growth velocities between 20 and 32 weeks of gestation is associated with lower birth weight percentiles and an increased risk of an adverse neonatal outcome.

## Methods

### Study design and participants

In this retrospective cohort study, we combined data from two electronic patient databases: the antenatal ultrasound database and the labour ward database of the Maastricht University Medical Centre (MUMC) to make an integrated database. We included women with singleton pregnancies without congenital anomalies, who delivered between April 2010 and July 2016 in the Maastricht University Medical Centre, and who had two available fetal growth scans, one between 18 and 22 weeks of gestational age and one between 30 and 34 weeks of gestational age. The pregnancies were dated according to the formula of Robinson calculated from crown-rump length (CRL) measurement in the first trimester [12]. We only included women, who had a delivery of a fetus with a birth weight between the 10th–80th centile. We excluded women with a birth weight below the 10th centile (SGA) and above the 80th centile (large-for gestational age). Patients were informed about research in this university medical centre and were given information about the data collection from their records for research purpose. Patients who refuse using their records, were excluded in this study. The study protocol was approved by the medical ethical committee of the Maastricht University Medical Centre (17–4-0.15.1/ab). All procedures were in accordance with institutional guidelines and adhered to the principles of the Declaration of Helsinki and Title 45, U.S. Code of Federal Regulations, Part 46, Protection of Human Subjects (revised 13 November 2001, effective 13 December 2001).

### Ultrasound growth examination

At the first hospital intake, data on length, weight, smoking status and medical and obstetric history, were collected and recorded in an electronic medical file. Body mass index was calculated by weight (kg) divided by height (m)-squared. An experienced sonographer recorded ultrasound measurements of abdominal circumference (AC), biparietal diameter (BPD), head circumference (HC) and femur length (FL), using a GE Voluson ultrasound machine, with a 2-5 MHz curved-array transducer, in accordance with the routine mid-trimester fetal ultrasound scan guidelines [13]. These fetal growth scans were performed in two periods, around 20 weeks (18–22 weeks) during the second trimester anomaly scan, and a second around 32 weeks (30–34 weeks) as a routine third trimester growth scan [14, 15]. Fetal growth parameters were recorded in Astraia electronic fetal medicine database (Astraia GMBH; Munich, Germany). Estimated fetal weight was calculated using the Hadlock equation [16][Hadlock C; log (10) BW = 1.335–0.0034(abdominal circumference [AC])(femur length [FL]) + 0.0316(biparietal diameter) + 0.0457(AC) + 0.1623(FL). For the purpose of this article we calculated velocities of each of the fetal growth parameters (in mm/week) as the difference in actual values (rather than percentiles) of the measured parameters between the two examination periods, divided by the number of weeks. Birth weight (core outcome) was transformed to percentiles according to the Dutch reference standard [17] with a birth weight between the 10th and 80th percentile considered to be appropriate-for-gestational-age (AGA). Further this AGA group was divided into two categories: 1) birth weight centiles 10–50 as the suboptimal appropriate-for-gestational-age (sAGA) group and 2) birth weight centiles 50–80 as the optimal appropriate-for-gestational-age group (oAGA).

In addition, we compared neonatal outcomes in two groups of neonates based on the premise that a fetus with an above average abdominal circumference at 20 weeks of gestation is “expected” to have an above average birth weight, and vice versa. Consequently, the first group consisted of neonates who had a below average birth weight despite an above average AC at 20 weeks (birth weight < expected), suggesting a late reduction in growth velocity, and the second group consisted of neonates who had a higher than average birth weight despite a below average AC at 20 weeks (birth weight ≥ expected).

### Neonatal data

Delivery and neonatal outcomes were registered by the obstetrician or in case of an adverse outcome by the paediatrician. Data about induction of labour and mode of delivery were collected. Mode of delivery was divided in subcategories; spontaneous vaginal, assisted vaginal, intrapartum- or prelabour caesarean. All deliveries were in the same hospital.

APGAR-scores were assessed at 1 and 5 min after birth. We used a cut-off of less than 5 at 5 min to determine adverse outcome [18]. Metabolic acidosis was defined as an umbilical artery blood pH < 7.0 and base deficit > 12 mmol/L. [19] The composite adverse neonatal outcome consisted of 4 complications: asphyxia, sepsis, respiratory distress syndrome [20] and transient tachypnea of the newborn [21]. These complications were recorded in medical files and were defined according to the judgments of the attending medical staff. Asphyxia was defined as intrapartum-related hypoxia-ischemia with multiple organ failure including encephalopathy [22]. Neonatal stay at the hospital was divided in general hospital stay and neonatal intensive care unit admission.

### Statistical analysis

The general characteristics were presented as mean and standard deviation (SD) for the total cohort and stratified by birth weight percentiles (50–80 as optimal appropriate-for-gestational-age (oAGA) group, and the birth weight percentiles 10–50 as suboptimal appropriate-for-gestational-age (sAGA) group).

Differences between the groups were calculated with the independent T-test for continuous variables and the Chi-square test for categorical variables. Analysis of variance (ANOVA) was used to compare growth velocities between different groups of birth weight percentiles. Post-hoc testing of differences was corrected for multiple testing using the Bonferroni-method. Growth velocities we reported using mean, SD and range. We used logistic regression analysis to estimate the association between abdominal circumference velocity and dichotomous neonatal outcomes, corrected for potential confounders. We considered maternal age, BMI and parity as potential confounding variables. All analyses were performed using SPSS Statistics 23 (IBM Corp, Armonk, NY). *P*-values of 0.05 or less were considered to indicate statistical significance.

## Results

### Participants

Between April 2010 and July 2016, we identified 7.720 pregnant women, with 28.649 fetal biometry scans. We excluded scans < 18 weeks of gestational age (*n* = 2.762), between 22 and 30 weeks of gestational age (*n* = 5.641) and > 34 weeks of gestational age (*n* = 3.789). After exclusion of twin pregnancies (*n* = 362), we had 7.348 singleton pregnancies in the labour ward database and 16.457 fetal biometry scans between 18 and 22 and 30–34 weeks of gestational age in the antenatal ultrasound database.

After cross-matching the two databases, women with growth scans outside the range of the intended periods or who delivered outside the MUMC hospital were excluded. Also, we excluded neonates with a birth weight percentile < 10 (*n* = 953) or birth weight percentile > 80 (*n* = 213), as well as those with intra-uterine fetal death (*n* = 18) and congenital anomalies (within 4 neonatal death) (*n* = 116).

**Fig. 1:**
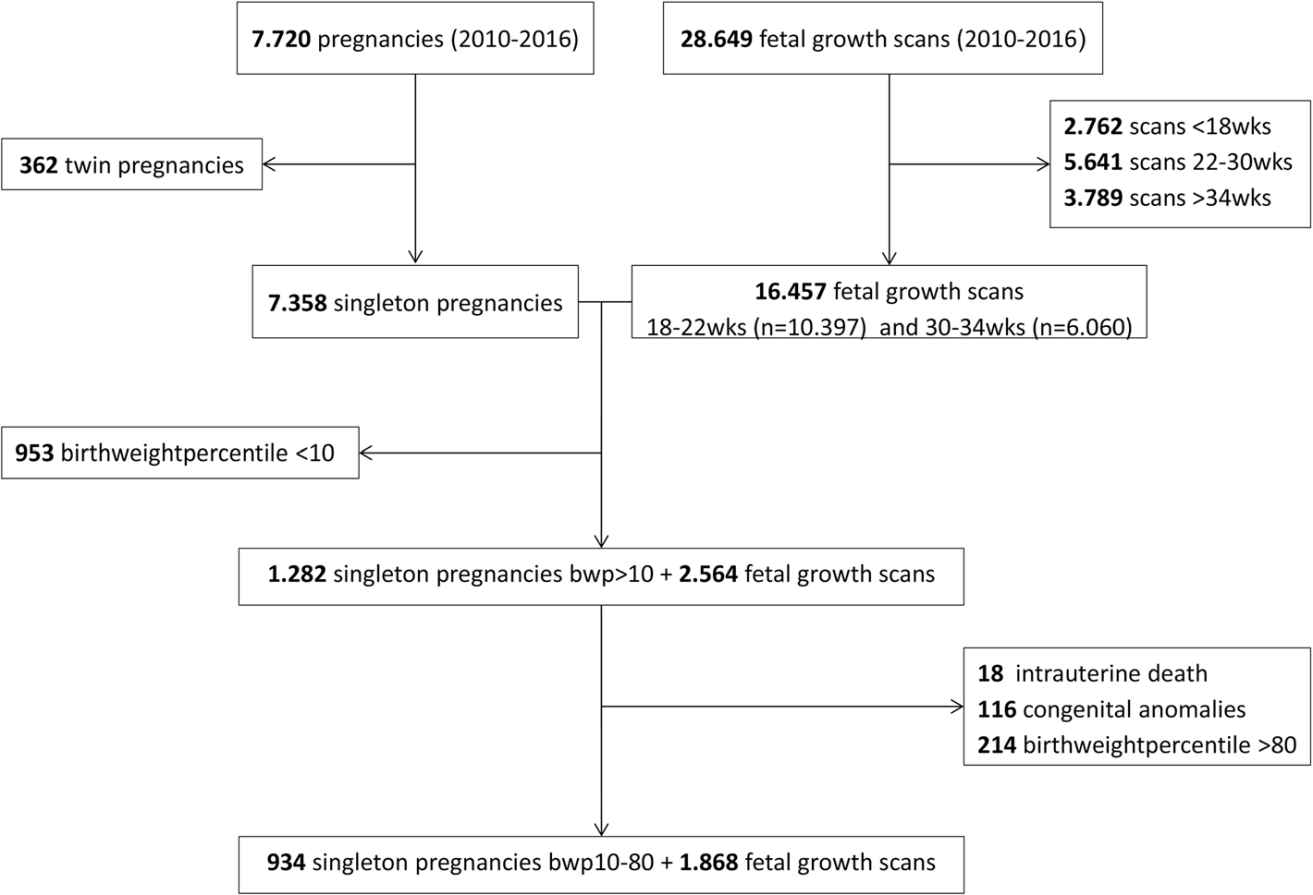
Flowchart of matched pregnancies and fetal growthscans in the appropriate-for-gestational-age (AGA) group

The final study cohort consisted of 934 singleton pregnancies (birth weight centiles 10–80) and 1.868 fetal ultrasound biometry scans (Fig. 1). The neonates were divided in categories: birth weight percentiles 10–50, considered as suboptimal appropriate-for-gestational-age (sAGA) (*n* = 569) and birth weight percentile 50–80, considered as optimal appropriate-for-gestational-age (oAGA) (*n* = 365).

The baseline characteristics of the study population are given in Table 1. Women had a mean age of 31.6 ± 5.1 years and a mean body mass index of 25.0 ± 5.7 kg/m2, 44.4% were primiparous and 11.8% were smokers. There were no statistically significant differences between the optimal and suboptimal AGA groups in baseline characteristics, and in gestational age at birth, 271.0 ± 13.7(212–295) days vs. 271.8 ± 12.2 (215–295) days (*p* = 0.35), except a lower percentage of ovulation induction (2.5% vs 0.7%) (*p* = 0.025). There was an obvious difference in birth weight (g) between the groups: 3425.4 ± 421.6 (1440–4125) in the optimal AGA vs. 3041.5 ± 402.3 (1353–3818) in the suboptimal AGA group (*p* < 0.001).

**Table 1:** Patient general characteristics of total study cohort with delivery details and stratified according to birth weight percentile category in oAGA reference group and sAGA group

Characteristic	Total cohort AGA (*n* = 934)	Optimal AGA (oAGA) Bwp50–80 (*n* = 365)	Suboptimal AGA (sAGA) Bwp10–50 (*n* = 569)	P
Maternal age (years)	31.6 ± 5.1(17–55)	31.6 ± 5.1 (17–55)	31.5 ± 5.1 (17–45)	0.816
Primiparous (%)	412 (44.4%)	160 (44.2%)	252 (44.6%)	0.904
Smoking (%)	89(11.8%)	27 (9.2%)	62 (13.6%)	0.067
Height (m)	1.67 ± 0.07(1.45–1.86)	1.68 ± 0.07(1.49–1.86)	1.67 ± 0.07 (1.45–1.86)	0.362
BMI (kg/m^2^)	25.0 ± 5.7(15.7–48.5)	25.5 ± 5.5(16.7–48.3)	24.7 ± 5.9 (15.7–48.5)	0.164
Mode of conception				
spontaneous	775 (91.7%)	335 (91.8%)	529 (93.0%)	0.501
Iui	13(1.5%)	4 (1.1%)	9 (1.6%)	0.536
Icsi	25(3.0%)	8 (2.2%)	17 (3.0%)	0.462
Ivf	19(2.2%)	9 (2.5%)	10 (1.8%)	0.454
Ovulation induction	13(1.5%)	9 (2.5%)	4 (0.7%)	0.025
GA at birth (days)	271.5 ± 12.8(212–295)	271.0 ± 13.7(212–295)	271.8 ± 12.2(215–295)	0.350
GA <34wks (n)	23 (2.5%)	13(3.6%)	10 (1.8%)	0.088
GA 34-36wks (n)	40(4.3%)	15(4.1%)	25(4.4%)	0.870
GA >36wks (n)	871(93.3%)	337 (92.3%)	534 (93.8%)	0.423
Birth weight (g)	3191.55 ± 450.6(1353–4125)	3425.4 ± 421.6(1440–4125)	3041.5 ± 402.3 (1353–3818)	< 0.001
Induction of labour (yes)	205 (21.9%)	89 (24.4%)	116 (20.3%)	0.168
Mode of delivery				
Spontaneous vaginal	722 (77.3%)	278 (76.2%)	444 (78.0%)	0.522
Assisted vaginal	124 (13.3%)	47 (12.9%)	77 (13.5%)	0.843
Intrapartum caesarean	32 (3.4%)	17 (3.0%)	15 (4.1%)	0.363
Prelabour caesarean	56 (6.0%)	25 (6.8%)	31 (5.4%)	0.399

Data are expressed as mean ± standard deviation (min-max) or *n* (%). *BMI* body mass index, *GA* gestational age

### Fetal growth velocities

**Fig. 2:**
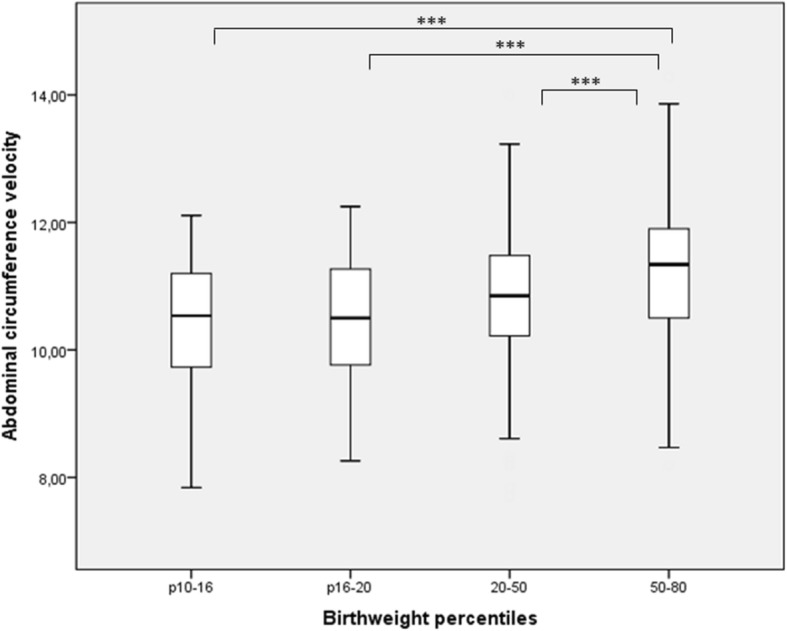
Boxplot of abdominal circumference velocity (mm/week) in the optimal appropriate-for gestational- age group (oAGA) (birthweight percentile 50–80) and birthweight percentiles 10–16, 16–20 and 20–50. Legend: *P*-values: * = *P* ≤ 0.05, ** = *P* ≤ 0.01, *** = *P* ≤ 0.001

Compared to oAGA neonates (BW p50–80), the sAGA group (BW p10–50) had reduced growth velocities across all studied parameters (in mm/week) (Table 2): abdominal circumference (10.72 ± 1.00 vs 11.23 ± 1.00, *p* < .001), head circumference (10.50 ± 0.80 vs 10.68 ± 0.77, *p* = 0.001), biparietal diameter (3.01 ± 0.28 vs 3.08 ± 0.27, *p* < .0001) and femur length (2.47 ± 0.21 vs 2.50 ± 0.22, *p* = 0.014), respectively. Within the sAGA neonates, the difference in growth velocity (in mm/week) was most prominent among the subgroup with the lowest birth weight percentiles (i.e. BW p10–16) for AC (10.45 ± 0.96, *p* < .0001), HC (10.26, *p* < .001) and BPD (2.91, *p* < .001) compared to the oAGA reference group (Fig. 2, only data for AC are shown). There was a linear relationship between AC velocity and birth weight (in gram, mm/week) (Birth weight = 1951.67 + 113.57 x AC velocity, *p* < .0001, R^2^ = 0.067).

**Table 2:** Ultrasound growth velocities (20–32 weeks, in mm/week) in the optimal appropriate-for-gestational- age (oAGA) and suboptimal appropriate-for-gestational-age group (sAGA)

	Optimal AGA (oAGA) group (n = 365) Bwp50–80	Suboptimal AGA (sAGA) group (*n* = 569) Bwp10–50	P
Abdominal circumference velocity	11.23 ± 1.00(8.19–14.28)	10.72 ± 1.00(7.70–14.00)	<.0001
Head circumference velocity	10.68 ± 0.77 (8.54–13.51)	10.50 ± 0.80 (6.79–12.67)	0.001
Biparietal diameter velocity	3.08 ± 0.27(2.31–3.99)	3.01 ± 0.28(1.96–3.85)	<.0001
Femur length velocity	2.50 ± 0.22(1.89–3.01)	2.47 ± 0.21(1.54–3.08)	0.014

Data are expressed as mean ± standard deviation (min-max)

### Neonatal outcomes

There were neither differences between the sAGA and oAGA in the frequency of induction of labour and the mode of delivery, nor in neonatal outcomes: composite adverse outcome, NICU and hospital stays, metabolic acidosis and APGAR5min < 5 (Table 3). However, using this composite adverse neonatal outcome as the independent variable to divide the cohort, neonates with an adverse composite outcome had significantly lower growth velocities (in mm/week) in abdominal circumference: 10.57 ± 1.33 vs 10.94 ± 1.00 (*p* = 0.034), head circumference 10.28 ± 0.99 vs 10.59 ± 0.79 (*p* = 0.003) and biparietal diameter 2.97 ± 0.30 vs 3.04 ± 0.27 (*p* = 0.043) compared to with neonates with normal outcome (Table 4).

**Table 3:** Neonatal outcomes in the optimal appropriate-for-gestational-age (oAGA) and suboptimal appropriate-for-gestational-age (sAGA) group

	Optimal AGA (oAGA) group (*n* = 365) Bwp50–80	Suboptimal AGA (sAGA) group (*n* = 569) Bwp10–50	P
Composite adverse neonatal outcome	25 (6.8%)	37 (6.5%)	*P* = 0.919
Hypoglycemia	23 (6.3%)	52 (9.1%)	*P* = 0.139
NICU stay (yes/no)	26 (7.1%)	35 (6.2%)	*P* = 0.588
Hospital stay (yes/no)	145 (39.7%)	226 (39.7%)	*P* = 0.998
Metabolic acidosis	14 (5.2%)	29 (6.5%)	*P* = 0.520
APGAR 5 min < =5	5 (1.4%)	6 (1.1%)	*P* = 0.759
APGAR 5 min < =3	0 (0%)	2 (0.6%)	*P* = 0.523

Data are expressed as *n* (%). Composite adverse neonatal outcome: asphyxia, sepsis, respiratory distress syndrome and transient tachypnoea of the newborn. NICU, neonatal intensive care unit. Metabolic acidosis, blood pH < 7.0 and base deficit > 12 mmol/L

**Table 4:** Ultrasound growth velocities (mm/week) in adverse and healthy neonatal outcome

	Composite adverse neonatal outcome (*n* = 62)	Healthy outcome (*n* = 872)	P
Abdominal circumference velocity	10.57 ± 1.33(7.70–13.86)	10.94 ± 1.00(7.84–14.28)	0.034
Head circumference velocity	10.28 ± 0.99(6.79–13.51)	10.59 ± 0.79(8.26–13.51)	0.003
Biparietal diameter velocity	2.97 ± 0.30(1.96–3.99)	3.04 ± 0.27(2.24–3.99)	0.043
Femur length velocity	2.46 ± 0.27(1.54–3.08)	2.48 ± 0.21(1.75–3.08)	0.579

Data are expressed as mean ± standard deviation (min-max)

There was an inverse association between the abdominal circumference velocity and a composite adverse neonatal outcome (−odds ratio (OR) = 0.667 (95%CI 0.507–0.879, *p* = 0.004), and between the abdominal circumference velocity and a neonate with NICU stay, (OR = 0.733 (95% CI 0.570–0.942, *p* = 0.015). There was no significant association between the abdominal circumference velocity and neonatal hospital stay, (OR = 0.915 (95%CI 0.805–1.039, *p* = 0.171), metabolic acidosis, (OR = 1.144 (95%CI 0.850–1.541, *p* = 0.374) and APGAR at 5 min < 5, (OR = 1.102 (95%CI 0.558–2.174, *p* = 0.780). All associations were corrected for maternal age, BMI and parity.

**Fig. 3:**
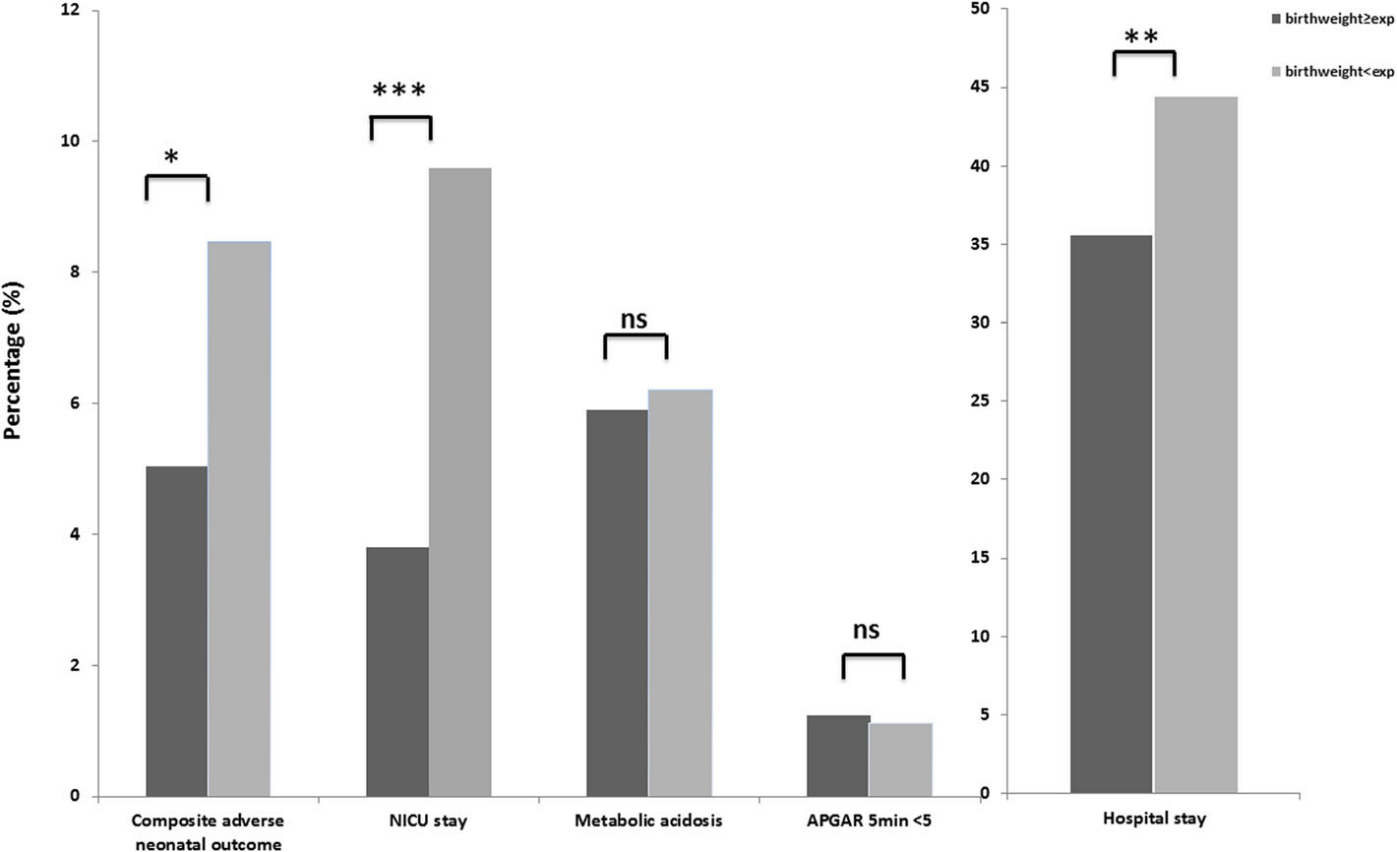
Bar chart of neonatal outcomes in birth weight ≥ expected and birth weight < expected. Legend: Data are expressed as *n* (%) Composite adverse neonatal outcome: asphyxia, sepsis, respiratory distress syndrome and transient tachypnoea of the newborn. NICU, neonatal intensive care unit, Metabolic acidosis, blood pH < 7.0 and base deficit > 12 mmol/L. Birth weight ≥ expected (dark bars), birthweight < expected (light bars). P-values are given, ns = not significant, * = *P* ≤ 0.05, ** = *P* ≤ 0.01, *** = *P* ≤ 0.001

In addition, neonates who had a below average birth weight despite an above average AC at 20 weeks (birth weight < expected), suggesting a late reduction in growth velocity had significantly more composite adverse neonatal outcomes (8.5% vs 5.0%, *p* = 0.047), more NICU stays (9.6% vs 3.8%, *p* = <.0001) and more hospital admissions (44.4% vs 35.6%, *p* = 0.006), compared to neonates who had a higher than average birth weight despite a below average AC at 20 weeks (birth weight ≥ expected) (Fig. 3).

## Discussion

### Main findings

In this retrospective study of non-anomalous singleton pregnancies, we investigated neonatal outcomes in a large cohort of neonates assumed to be ‘appropriate-for-gestational-age’ (AGA) (birth weight centiles between 10 and 80) in relation to their antenatal growth velocities. We aimed at identifying a subgroup within AGA neonates with reduced growth velocity, but who were not classified as small or growth restricted as their birth weight was above the 10th centile according to the Dutch perinatal registry system. In this study, these AGA neonates with suboptimal birth weight (arbitrarily defined as birth weight p10–50) had reduced velocities of all fetal growth parameters compared to the optimal AGA reference group (birth weight p50–80). Although we could not detect significant differences in neonatal outcomes based on birth weight categories, neonates with a composite adverse neonatal outcome and NICU stay had significantly lower growth velocities in the abdominal circumference, head circumference and biparietal diameter velocity compared with healthy neonates. These data suggest that abnormal fetal growth velocities are associated with adverse neonatal outcomes of presumed appropriate-for-gestational-age neonates due to suboptimal fetal growth.

### Strengths and limitations

Strengths of this study were the large sample size and the comprehensive registration of ultrasound, obstetric and neonatal parameters in a single tertiary centre. Another strength of this study is the introduction of a new and practical method of assessing fetal growth potential within the AGA group using a simple calculation that can be readily employed and interpreted in low-resource setting without the need of complex mathematical models. An important limitation of the study is the retrospective design, with the inherent risk of selection bias and the exclusion of many cases in which the ultrasound examination did not fall within the specified time periods. Another limitation is the heterogeneity of the study population with various risk profiles for IUGR, as the study was performed in a tertiary referral centre. There was inadequate recording of determinants of obstetric risk and other confounders (such as ethnicity) to enable post-hoc stratification. This might limit the interpretation of our data and the generalizability to an unselected low-risk population. Caution must also be taken in interpreting our data as the association of fetal growth velocities with neonatal birthweight and adverse outcome does not necessarily imply the ability of such ultrasound parameters to identify individuals at risk for adverse outcomes. The degree of overlap in the min-max range of these velocities between normal and abnormal groups, especially in a large sample as in this study, weakens the predictive power of such parameters [23].

### Interpretation

Undiagnosed fetal growth restriction is recognized as a major contributor to stillbirths and perinatal morbidity [24, 25]. Traditionally, the use of the 10th birth weight percentile to define SGA (as a proxy to neonates with growth restriction) has failed to reduce perinatal morbidity in the past decades, despite major improvement in neonatal and obstetric care [7]. This is undoubtedly related to failure to recognize AGA fetus who are ‘at risk’ of adverse outcome because of suboptimal growth by current standards [6]. We excluded SGA neonates (birth weight the 10th centile) from the analysis, as this group already receives enough attention, although it is tempting to speculate that fetal growth velocities are reduced even further in severe FGR compared to constitutionally small neonates. In an effort to include only fetus with normal growth velocity, we set up the upper cut-off value for AGA neonates at the 80th centile. Although LGA neonates is traditionally defined as BW > 90th centile, neonates with BW between 80-90th centile could potentially include fetus with pathologically accelerated growth due to e.g. gestational diabetes.

Determining optimum fetal growth is the cornerstone of proposing strategies for antenatal prediction and surveillance of pregnancies at risk of fetal growth restriction. Alas, this is an intricate and complex concept with competing and interacting influences from genetic, epigenetic and environmental backgrounds [26]. Several authors addressed this topic in reviews, editorials and observational trials, proposing different methods to identify fetus who fail to maintain their growth trajectory, such as individualized growth assessment (IGA) [11, 27], AC velocity [28], AC z-score [15] or conditional AC [14, 29]. In this study, although, all ultrasound parameters of fetal growth were reduced in suboptimal AGA neonates and those with an adverse neonatal outcome, we propose AC velocity as a practical and interpretable parameters to use in future studies. Abdominal circumference is the most appropriate ultrasonic measurement for the prediction of growth restriction in high-risk subjects [30], it is a specific marker for small-for-gestational-age fetusses [31] and, in the lowest decile, it can be used to predict adverse neonatal outcome [15]. We also discuss using the term “suboptimal appropriate-for gestational-age”(sAGA) to define those fetus who have possibly failed to reach their genetically predetermined growth potential. The available methods to chart fetal growth show considerable differences [32] and there is no consensus among authorities on the best chart to implement. The INTERGROWTH-21st study formulated ultrasound-based, universally fetal growth charts, corrected for different countries and ethnicities, and the World Health Organization published international fetal growth charts, based on multinational data [33]. However, both charts reflect population standards rather than individualized growth standards for a particular fetus/neonate, and a more accurate approach would consider each fetus as its own individual control by computing growth potential based on an early growth trajectory. Comparing actual birth weight or ultrasound parameters of fetal growth to this expected individualised growth standard was shown to correlate to neonatal outcome and placental pathology and can potentially improve the differentiation between true FGR and constitutional smallness [10, 11, 34]. Future studies comparing these different strategies should deliver the verdict on which standards to be followed internationally for an improved and modern definition of normal and abnormal fetal growth.

This study highlights the importance of performing a third trimester biometry scan in an unselected population to determine target fetal growth. In the Netherlands, third trimester biometry scans are scheduled only for women who are at high risk of fetal growth restriction, according to national guidelines and agreements between gynaecologists and primary care midwives. There is ample evidence suggesting that the detection rate of small-for-gestational age improves with the additional scan in the third trimester [35] and triples the detection of neonatal morbidity in small-for-gestational age neonates [15]. An additional scan in appropriate-for-gestational-age neonates gives more insight in an eventually slowing fetal growth trajectory, which can be used as an indicator for fetal well-being [36]. It is unclear, however, when a third trimester biometry scan should be scheduled to improve the predictive power for adverse outcomes [28]. The addition of doppler measures of fetal haemodynamic compensation and biochemical measures of placental function to third trimester biometry scan is proposed to improve the performance of these tests for fetus at risk of intrapartum compromise and asphyxia [37–40].

## Conclusion

In conclusion, neonates with ‘appropriate’ birth weight for gestational age are a heterogeneous population, that includes a small ‘at risk’ group of fetuses who failed to reach their growth potential. How to tease out these apparently normal grown but ‘relatively small for gestational age’ fetus is a major challenge. Measuring abdominal circumference growth velocity in the third trimester, in combination with fetal dopplers and biomarkers of placenta function, could improve the identification of at risk fetus and are potential candidates for inclusion in multivariate models predicting fetal growth restriction.

## Abbreviations

AC: Abdominal circumference
AGA: Appropriate-for-gestational-age
BPD: Biparietal diameter
EFW: Expected fetal weight
FGR: Fetal growth restriction
FL: Femur length
HC: Head circumference
IUGR: Intrauterine growth
NICU: Neonatal intensive care unit
SGA: Small-for-gestational-age
